# Study protocol for a randomized controlled trial comparing the effectiveness of physical exercise and melatonin supplement on treating sleep disturbance in children with autism spectrum disorders

**DOI:** 10.1371/journal.pone.0270428

**Published:** 2022-07-06

**Authors:** Andy Choi Yeung Tse, Paul Hong Lee, Esther Yuet Ying Lau, James Ching Hei Cheng, Amy Wing Yin Ho, Elvis Wing Him Lai

**Affiliations:** 1 Department of Health and Physical Education, Education University of Hong Kong, Hong Kong, China; 2 Department of Health Sciences, University of Leicester, Leicester, United Kingdom; 3 Department of Psychology, Education University of Hong Kong, Hong Kong, China; 4 Department of Paediatrics and Adolescent Health, United Christian Hospital, Hong Kong, China; 5 Department of Chemical Pathology, Chinese University of Hong Kong, Hong Kong, China; 6 Department of Psychiatry, The Hong Kong Castle Peak Hospital, Hong Kong, China; Prince Sattam Bin Abdulaziz University, College of Applied Medical Sciences, SAUDI ARABIA

## Abstract

**Background:**

Previous study showed that both melatonin supplement and physical exercise intervention could improve sleep quality in children with autism spectrum disorders (ASD) with the increase in endogenous melatonin level. However, none of the studies have directly compared the effectiveness between the two interventions on treating sleep disturbance in children with ASD. Without direct comparison, we do not know which intervention is better. Thus, we designed a study to compare which intervention is more effective to treat sleep disturbance in children with ASD and to examine whether the combination of the two could be the most efficacious. We present a protocol for conducting a randomized controlled trial to compare the effectiveness of physical exercise and melatonin supplement on treating sleep disturbance in children with ASD.

**Study design:**

The proposed study will be a four-group randomised control trial (RCT) design, with equal allocation of participants to the three intervention groups and one control group.

**Methods:**

All eligible participants will be randomly allocated to a morning jogging group, a melatonin supplement group, a combination group and a control group. Changes in sleep quality will be monitored through actigraphic assessment and parental sleep logs. Melatonin levels represented by 6-sulfoxymelatonin will be measured from the participants’ 24-h and the first morning void urinary samples. All the assessments will be carried out before the intervention (T1), in the mid of the study (5 weeks after the commencement of the study) (T2) and after the 10-week intervention (T3). Level of statistical significance will be set at 5% (i.e. *p* < .05). The results of this trial will be submitted for publication in peer-reviewed journal.

**Findings:**

The findings will provide evidence to determine whether physical exercise or melatonin supplement or the combination of interventions is the most effective to treat sleep disturbance in children with ASD.

## Introduction

Autism spectrum disorder (ASD) is a neurodevelopmental disorder. In Hong Kong, the latest prevalence rate is 1.5% and the rate is predicted to increase [1]. Children with ASD are characterized, in varying degree, by deficits in social communication and social interaction, restricted and repetitive behavior, interests and activities. Moreover, children with ASD are also more susceptible to sleep disturbance than children with typical development (see Elrod et al. [2] for review). Common sleep disturbances in children with autism include delayed sleep onset, difficulty in sleep maintenance and insufficient sleep duration [2]. Given the importance of adequate sleep in child development, sleep disturbance in children with ASD is detrimental. Research shows that poor sleep is closely related to poor cognitive performance [3] (e.g., impaired learning performance, memory consolidation) and intensified behavioral problems [4] (e.g. increased stereotypic behavior) among children with ASD, which subsequently worsen quality of life of ASD individuals and their families [4]. As a result, developing effective intervention strategies are crucial.

To achieve so, we need to understand the causes of sleep disturbance in the population. Over decades, a growing body of literature has shown that physiological differences in children with ASD may be the major cause of sleep problems in children with ASD (see Maxwell-Horn et al. [4] for review). For example, children with ASD appear to have an increased state of physiological arousal than their TD peers [5], which is evidenced by higher heart rates during sleep, increased skin conductance at rest condition and after auditory stimulation, as well as lower melatonin production than their TD counterparts [6]. Currently, parent-directed behavioral sleep interventions (e.g., extinction, fade bedtime) are recommended as first-line treatment for sleep disturbance in ASD [7]. However, these types of intervention are very intensive and may sometimes be very stressful to both children and parents (e.g. extinction intervention [8]). Moreover, evidence for the intervention efficacy remains largely inconclusive due to small sample sizes and lacked follow-up data on sustainability [9]. If the behavioral intervention does not improve the problem, then the use of pharmacologic treatment is generally considered by clinician and parents. Given the decreased melatonin secretion as mentioned earlier, melatonin supplement—a nutritional supplement with sleep-promoting and sleep phase shifting properties, has gained widespread public acceptance as an alternative to FDA-approved medications due to its favourable side-effect profile and relatively low cost [10]. Several meta-analyses have also provided supportive evidence that melatonin supplement positively impacts sleep in children with autism, including reduced sleep onset latency, increased total sleep duration and improved sleep efficiency [11]. Despite favourable evidence on the use of melatonin supplement, it is still wise to “start low and go slow” when turning to this intervention [5], particularly given reports of adverse events such as somnolence, headache, daytime drowsiness and fatigue in some children [7]. Therefore, researchers are exploring other non-behavioral and non-prescription pharmaceutical approaches with undesirable side effects to treat sleep problems in children with ASD. One intervention that has received growing attention is physical exercise.

Over the past decade, a significant body of research has investigated the effects of physical exercise on sleep and the results generally supported the positive impact of physical exercise on sleep (see Kelley et al. [12] for review). Previous studies also support the sleep benefits of physical exercise in children with ASD. For example, we have examined the impact of a basketball training intervention on sleep quality (sleep efficiency and wake after sleep onset) and cognition (inhibition control and working memory) in 40 children with ASD [13]. Research revealed a significant improvement in sleep efficiency and reduction in wake after sleep onset time in the intervention group but not in the control group [13]. Similar sleep benefits following physical exercise were also evident in a more recent study [14], where we showed that jogging intervention had significantly improved sleep efficiency and sleep duration in children with ASD. More remarkably, we also observed that the endogenous melatonin level, as reflected by the 6-sulfatoxymelatonin (aMT6s), was significantly increased within the jogging intervention group, while no change was found in the control group. Physical exercise appears to be capable of modulating the endogenous melatonin level in children with ASD, which in turns improves their sleep quality. If it is so, it is then natural to ask which intervention (i.e. physical exercise or melatonin supplement of the combination of two) is more effective in treating sleep disturbance improving the sleep quality of children with ASD. Without direct comparison, this question remained unanswered. Therefore, here we propose a new study protocol.

This study protocol has two objectives: (1) to determine which intervention is more effective in improving sleep quality in children with ASD and 2) to explain how these interventions impact on sleep via melatonin-mediated mechanism model. We hypothesise that the combination intervention (i.e. jogging and melatonin supplement) is the most effective because it may provide sufficient melatonin level to enhance sleep quality through both physical exercise and melatonin supplement.

## Methods/Design

### Study design

The proposed study will be a single blinded four-group randomised control trial (RCT).

### Participants

Participants will be screened using the following inclusion criteria: (1) clinical diagnosis of ASD by a physician based on the Diagnostic and Statistical Manual of Mental Disorders, 5th edition, (DSM-5) [15]; (2) age 8–11 years; (3) pre-puberty as indicated by Tanner stage I through screening by a physician (to prevent any puberty influence on hormonal response); (4) being given an average of 8 hours of sleep per night by their parents over the past 3 months; (5) parents reported sleep onset delays of 30 minutes of longer on three or more nights per week; (6) free of psychotropic medications (allergy medications and medications for constipation are allowed); (7) non-verbal IQ over 50 using a brief version of Wechsler intelligence scale for children (Chinese revised, C-WISC) [16] (since lower IQ is liked to higher prevalence of comorbid psychiatric disorders and seizures); (8) Social Response Scale T-score 80 or above (to control for the ASD severity) and (9) no concurrent medication for at least three months before the study or any prior melatonin treatment.

Exclusion criteria are: (1) with one or co-morbid psychiatric disorders; (2) with other medical conditions that limit their physical exercise participation and sleep (e.g., asthma, seizure, cardiac disease etc); and (3) with a complex neurologic disorder (e.g., epilepsy, phenylketonuria, fragile X syndrome, tuberous sclerosis) and (4) participants who are currently meeting physical exercise guidelines (i.e. 60 minutes of moderate and vigorous physical exercise each day).

### Intervention

**Fig 1:**
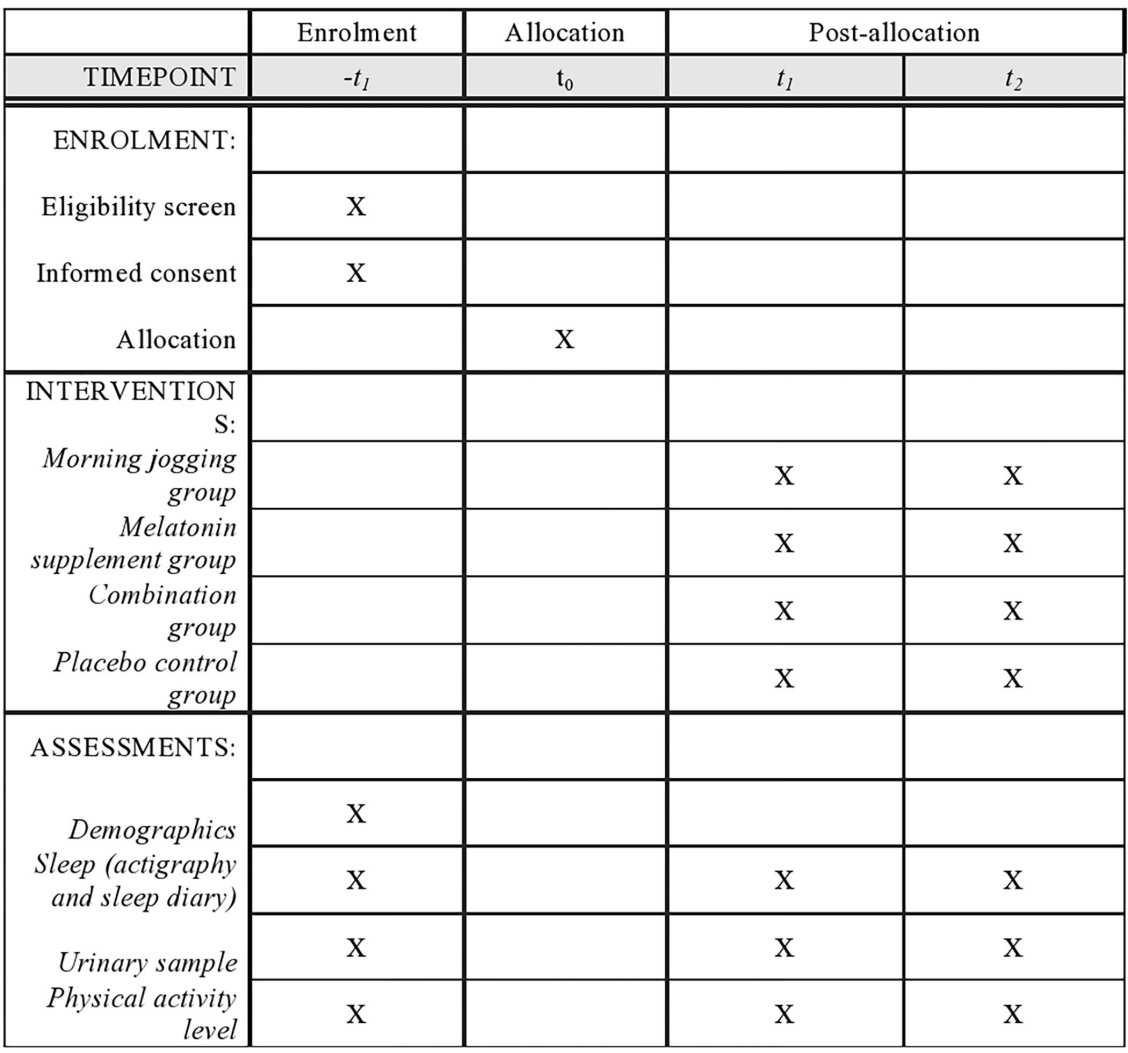
SPIRIT schedule of enrolment, interventions, and assessments of the study.

**Fig 2:**
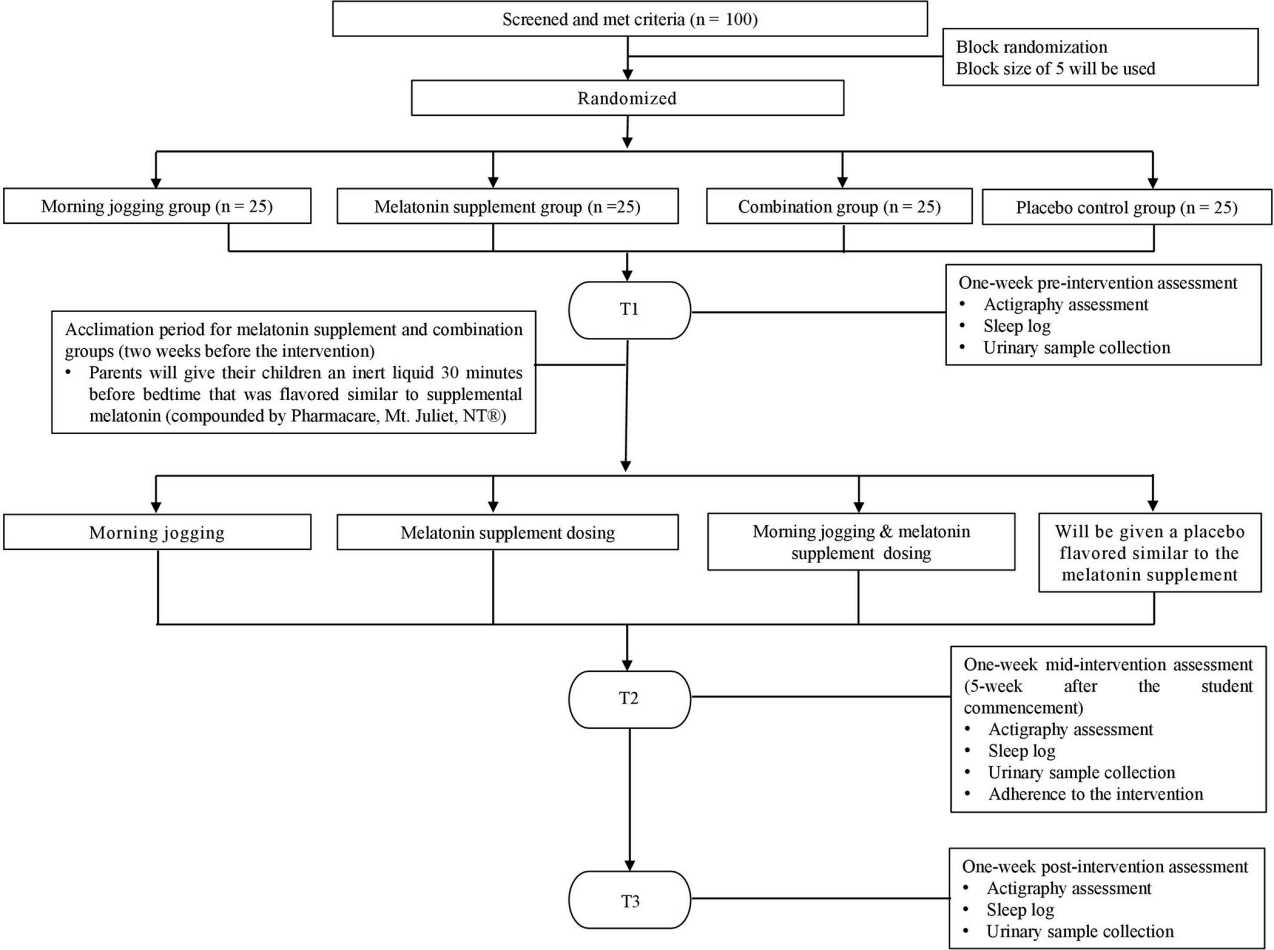
Flow diagram of the study.

Each participant will attend 3 one-week-long assessments in their respective schools, where we will assess their habitual sleep patterns and endogenous melatonin level before the intervention (T1), in the mid of the study (5 weeks after the commencement of the study) (T2) and after the 10-week intervention (T3). The mid-assessment is valuable to know how much variance in sleep behaviors is accounted by variance in melatonin, as well as to assess the adherence of the interventions. SPIRIT schedule of the study and a flow diagram of the study are shown in Figs 1 and 2 respectively. The estimated starting date of the study will be 1^st^ August, 2022 and the estimated ending date will be 30^th^ June, 2023.

#### Intervention A—Morning jogging group

The training protocol, assessment and instructional technique will be referenced on our previous protocol in children with ASD [17]. However, we will modify the frequency of the jogging sessions to suit the comparison with other intervention group (i.e. intervention B and C). This intervention will be a 10-week jogging program consisting of 50 sessions (5 sessions per week, 30 min per session) in each participating school. The increase of jogging sessions from the previous protocol [17] is to make the prescription equivalent to the melatonin supplement prescription for other interventions (B and C), which will be prescribed to the participants every day, according to the previous protocol [17]. Moreover, this is more close to the World Health Organization (WHO) recommendation that everyone should participate in physical exercise daily. Meanwhile, the jogging program is confined to morning sessions based on the favourable sleep outcomes from previous study [18]. To counteract the possible influence of natural sunlight exposure, all jogging sessions will be confined to indoor setting. Each intervention session will be administered by a trained research assistant assisted by student helpers. All the research staff (i.e. student helpers and research assistant) will be required to attend a workshop to standardize the procedure on implementing the intervention, as well as handling and motivating their ASD partners. Each session will be conducted in an identical format with 5 minutes of warm-up activities, followed by 20 minutes of jogging (intervention), and 5 minutes of cool-down activities. In the jogging activity, participants will be asked to jog side-by-side with the research staff around an activity circuit (57m x 50m) marked with 4 red cones. Participants are instructed to jog at a moderate intensity level. The intensity level of jogging will be measured by heart rate monitor (Polar H1). Considering the general low physical fitness of children with ASD, physical exercise with a heart rate above 50% of the maximum heart rate (subtracting the participant’s age from 220) is regarded as moderate intensity. Motivation techniques (e.g. verbal cue, visual chart, favourite post-intervention activity) will be used to enhance the compliance rate. Meanwhile, questionnaire will be given to the research staff assisting the jogging intervention to assess the adherence of the intervention at T2. The feasibility, community-viability and sustainability of the exercise intervention are confirmed with previous research experience and the cooperation of the participating schools. Since these intervention sessions are conducted in the morning hours before school start times, the participants may get less sleep time since they must arrive at school early enough to do the exercise. To ensure the participants to have sufficient sleep, they are strongly encouraged to go to bed earlier at night.

#### Intervention B—Melatonin supplement group

Participants in this intervention group will undergo a 10-week melatonin supplement intervention period, where melatonin supplement (Natrol®, Chatsworth CA) will be provided 30 minutes before bedtime [19]. The prescription time (i.e. 30 minutes before bedtime) and the dosage of 3mg will be used because these are optimal for most of the participants as suggested by Malow and colleagues [19]. 1 mg and 9 mg are not suggested to ensure the effectiveness of the intervention while preventing the potential daytime sleepiness. Similar to the aforementioned intervention, adherence of the intervention will be assessed at T2.

#### Intervention C—Combination group

Participants will receive the jogging program and supplemental melatonin dose with identical format as that in the intervention A and B (e.g., identical duration, identical manpower, identical warm-up and cool-down, identical dose, identical acclimation procedure before the intervention adherence of the intervention for this group will also be assessed at T2.

#### Placebo control group

Participants in the control group will receive no jogging and melatonin supplement dosing activity. However, they will be given a placebo flavored similar to the melatonin supplement (compounded by Pharmacare, Mt. Juliet, NT®). Meanwhile, they will also be required to wear an actigraph to control for their physical activity level at the assessment points (i.e. T1, T2, and T3). They will be expected for following their daily routine without participating in any additional formal physical exercise training program throughout the whole study period (T1-T3). After T3, they will be assisted with jogging program to recognize their contribution as controls.

To ensure all the interventions are delivered as intended, regular meetings among the research team will be hosted by the PA to monitor the progress and to discuss any difficulty encountered. Meanwhile, each participating family will be provided with a financial incentive, which is a good strategy evidenced by a high compliance rate in our ongoing study.

### Study measures

The demography and developmental history of each participant will be obtained from their parents or schools. And the following measurements will be carried out at T1, T2 and T3.

#### Primary outcome measures

*Sleep*. Four sleep parameters including sleep onset latency (length of time taken to fall asleep, expressed in minutes, SOL); sleep efficiency (actual sleep time divided by time in bed, expressed as a percent, SE); wake after sleep onset (length of time they were awake after sleep onset, expressed in minutes, WASO) and sleep duration (total sleep in hours and minutes, SD) will be objectively measured by a GT3X accelerometer [19]. Participants will be asked to wear the device on the non-dominant wrist for seven consecutive days (Monday to Sunday). Non-wear time is defined as 60 min of consecutive zeros with a 2-min spike tolerance [19, 20]. The night (2200–0700) will be considered invalid if the wear time is less than 8 h and will be excluded from the analysis. In addition, participants’ sleep patterns will be logged by their parents using Children’s Sleep Habits Questionnaire–Chinese version (CSHQ-C) [21], which is a validated 45-item parent-administered questionnaire to examine sleep patterns of young children including children with ASD [21]. Moreover, parents will be also asked to log the bedtime, sleep latency, sleep start, sleep end, wake-up time and assumed sleep length in a sleep log for the whole assessment week.

*Endogenous melatonin level*. The endogenous melatonin level of the participants, reflected by the 6-sulfatoxymelatonin (aMT6s, creatinine-adjusted morning urinary melatonin representative), will be measured from their 24-h and first morning urinary samples during weekend and Monday respectively [14], and the participants will be required to stay at home for sample collection. All urine samples will be collected using 24-h and first morning void urine bottles. Once they have been collected, the research staff will immediately collect the samples and they will be stored at −80°C before analysis.

#### Secondary outcome measure

*Physical activity level*. The physical activity level of the participants will also be measured as secondary data to examine its relationship to cognitive functions as suggested by previous studies [22, 23]. It will be measured using accelerometers (i.e. GT3X). The data used for analysis are the times spent in sedentary activity and MVPA based on the default energy expenditure algorithm in the accelerometer device [24]. The day (0700–2100) will be considered invalid if the wear time is less than 10 hours and will then be excluded from the analysis.

### Sample size calculation

Previous study [13, 14] showed physical exercise had notable effects (corresponding to a Cohen’s *d* of about 0.9) on improving sleep onset latency, sleep duration and sleep duration. We assumed that melatonin supplements have similar effects on sleep. A sample of 20 participants per group is computed by G*power software to achieve a power of 80% and a level of significance of 5% and 95% confidence level. Assuming 25% attrition rate, 25 participants per group will be recruited (i.e. the total number is 100).

### Randomization

Participants will be randomly assigned to three intervention groups or a placebo-control group by a trained research staff using MS Excel randomization function. Block randomization with block size of 5 will be used for equal allocation ratios.

### Patient and public involvement

Patients and public will not be involved in the study.

### Blinding

The person analysing the sleep parameters and melatonin level will be blinded for the group assignment.

### Ethical considerations

Prior to the study, information about the study were provided to all participants and their parents. Written consent and verbal assent were obtained from them. All data were encrypted with passwords and only the authors could access to the data. Ethical approval was obtained from the first author’s institution (Reference no: 2019-2020-0470). The results of the study will be published in a peer-reviewed journal. Findings of the study will also be shared with other university and non-governmental organisations in Hong Kong that specialise in autism by means of a formal dissemination webinar. The trial registration number is NCT04878198 from https://clinicaltrials.gov/.

### Statistical methods

All statistical analyses will be conducted using SPSS (version 26.0) for Windows (SPSS Inc., Chicago, IL, USA). All the data will be entered into SPSS by the research assistant. One-way (4 Groups: physical exercise vs melatonin supplement vs combined exercise and melatonin supplement vs placebo-control) analysis of variances (ANOVA) with repeated measures will be performed to compare changes in actigraphy data and endogenous melatonin level between and within groups over different time points (i.e. T1-T3). Post-hoc analyses will be performed when any significant difference both between- and within-group is found in any of the outcome variables. Bonferroni correction will be used to adjust the alpha levels. Depending on the proportion of missing data and the assumption of normality, multilevel regression or a generalized estimating equation (GEE) will also be used as a sensitive analysis to assess the effects of the physical exercise intervention, melatonin supplement, time effect, and their interaction with sleep outcomes and melatonin level outcomes if there is any missing data. Average time spent in both daily sedentary activity and MVPA at baseline will be used as covariates. Linear regression will be used to analyse the association between participant’s adherence to the intervention and the outcome variables as a secondary analysis. Mediation analysis will be conducted to examine the mediating effect of endogenous melatonin level at T2 on the relation between physical exercise and sleep at T3. Preacher and Hayes’ bootstrapping and conditional process modeling will be used to determine the significance of the mediating effect. A p-value of <0.05 will be considered statistically significant. Both proposal analysis and intention-to-treat analysis will be carried out. Demographic variables (e.g. age, sex, BMI) will be adjusted if they demonstrate clinically significant prognostic effects on the outcomes (with absolute correlation or Cohen’s d effect size of >0.2) for both main analysis and mediation analysis.

## Discussion

This study protocol is designed largely based on our previous protocol and study [14, 17] that showed urinary melatonin level in children with ASD could be affected by their physical exercise participations. The study takes one-step forward by further comparing the efficacy of physical exercise and melatonin supplement intervention on treating sleep disturbance in children with ASD. The findings of the study will have three significant impacts. First, if the three interventions were equally effective, then physical exercise, which is more naturalistic and brings other health benefits, can be an alternative option for practitioners and parents to treat sleep problems for their patients and children with ASD. Second, if there was a difference between the three interventions on treating sleep problems, it will then inform further research on the relationship between physical exercise, melatonin supplement and sleep. For example, if the combination was the most effective intervention to ameliorate the sleep disturbance in children with ASD, it would imply researchers may need to further investigate other factors such as neurological factors (e.g. brain derived neurotrophic factor) or psychological factors (e.g. happiness, motivation) that may play a role on mediating the relationship between physical exercise and sleep. Third, this study enables the investigators to follow up their previous study, where they were investigating the melatonin-mediated mechanism between physical exercise and melatonin. The findings of this study will allow the investigators to reaffirm if melatonin mediates the relation between physical exercise and changes in sleep quality, which would further strengthen the current limited evidence on the efficacy of physical exercise for sleep problems in children with ASD. The findings of the proposed study will ultimately lead to optimal treatment interventions for sleep disturbance not only in children with ASD, but also in any population suffering from sleep disturbances.

## Supporting information

S1 Checklist (DOC)

S1 File (PDF)

## References

[1] Global Autism Population. Autism Hong Kong. http://www.autism.hk/2019/asd-pop.htm. Accessed 11th November, 2021.

[2] Elrod MG, Hood BS. Sleep differences among children with autism spectrum disorders and typically developing peers: A meta-analysis. J Dev Behav Pediatr. 2015;36(3):166–177. doi: 10.1097/DBP.0000000000000140 25741949

[3] Maski K, Holbrook H, Manoach D, Hanson E, Kapur K, Stickgold R. Sleep dependent memory consolidation in children with autism spectrum disorder. Sleep. 2015;38(12):1955–1963. doi: 10.5665/sleep.5248 26194566 PMC4667378

[4] Maxwell-Horn A, Malow BA. Sleep in autism. Semin Neuro. 2017;37(4):413–418. doi: 10.1055/s-0037-1604353 28837988

[5] Harder R, Malow BA, Goodpaster RL, et al. Heart rate variability during sleep in children with autism spectrum disorder. Clin Auton Res. 2016;26(6):423–432. doi: 10.1007/s10286-016-0375-5 27491489 PMC5106315

[6] Gringras P, Nir T, Breddy J, Frydman-Marom A, Findling RL. Efficacy and safety of pediatric prolonged-release melatonin for insomnia in children with autism spectrum disorder. J Am Acad Chil Adolesc Psychiatry. 2017;56(11):948–957. doi: 10.1016/j.jaac.2017.09.414 29096777

[7] Weiskop S, Richdale A, Matthews J. Behavioural treatment to reduce sleep problems in children with autism or fragile X syndrome. Dev Med Child Neurol. 2005;47(2):94–104. doi: 10.1017/s0012162205000186 15707232

[8] Durand VM, Christodulu KV. Description of a sleep-restriction program to reduce bedtime disturbances and night waking. J Pos Behav Interv. 2004;6(2):83–91.

[9] Malow BA, Byars K, Johnson K, et al. A practice pathway for the identification, evaluation, and management of insomnia in children and adolescents with autism spectrum disorders. Pediatrics. 2012;130(Supplement 2):S106–S124. 23118242 10.1542/peds.2012-0900I PMC9923883

[10] Abdelgadir IS, Gordon MA, Akobeng AK. Melatonin for the management of sleep problems in children with neurodevelopmental disorders: a systematic review and meta-analysis. Arch Dis Child. 2018. doi: 10.1136/archdischild-2017-314181 29720494

[11] Rossignol DA, Frye RE. Melatonin in autism spectrum disorders: a systematic review and meta-analysis. Dev Med Child Neurol. 2011;53(9):783–792. doi: 10.1111/j.1469-8749.2011.03980.x 21518346

[12] Kelley George A, Kelley Kristi S. Exercise and sleep: a systematic review of previous meta-analyses. J Evid Med. 2017;10(1):26–36. doi: 10.1111/jebm.12236 28276627 PMC5527334

[13] Tse ACY, Lee PH, Chan KSK, Edgar VB, Wilkinson-Smith A, Lai WHE. Examining the impact of physical activity on sleep quality and executive functions in children with autism spectrum disorder: A randomized controlled trial. Autism. 2019;23(7):1699–1710. doi: 10.1177/1362361318823910 30663324

[14] Tse ACY, Lee PH, Zhang J, Chan RCY, Ho AWY, & Lai WHE. Effects of exercise on sleep, melatonin level and behavioral functioning in children with autism. Autism. 2022. (Epub ahead of print). 10.1177/13623613211062952 35083939

[15] Weitlauf AS, Gotham KO, Vehorn AC, Warren ZE. Brief report: DSM-5 "levels of support:" a comment on discrepant conceptualizations of severity in ASD. J Autism Dev Disord. 2014;44(2):471–476. doi: 10.1007/s10803-013-1882-z 23812664 PMC3989992

[16] Gong YX, Cai TS. The Wechsler intelligence scale for children revised in china (C-WISC). Hunan Maps Press; 1993.

[17] Tse ACY, Lee PH, Zhang J, Lai EWH. Study protocol for a randomised controlled trial examining the association between physical activity and sleep quality in children with autism spectrum disorder based on the melatonin-mediated mechanism model. BMJ Open. 2018;8(4). doi: 10.1136/bmjopen-2017-020944 29654045 PMC5905756

[18] Pan C-Y, Chu C-H, Tsai C-L, Sung M-C, Huang C-Y, Ma W-Y. The impacts of physical activity intervention on physical and cognitive outcomes in children with autism spectrum disorder. Autism. 2017;21(2):190–202. doi: 10.1177/1362361316633562 27056845

[19] Malow BA, Adkins KW, McGrew SG, et al. Melatonin for sleep in children with autism: A controlled trial examining dose, tolerability, and outcomes. J Autism Dev Disord. 2012;42(8):1729–1737. doi: 10.1007/s10803-011-1418-3 22160300 PMC3368078

[20] Wachob D, Lorenzi DG. Brief report: Influence of physical activity on sleep quality in children with autism. J Autism Dev Disord. 2015;45(8):2641–2646. doi: 10.1007/s10803-015-2424-7 25791123

[21] Li SH, Jin XM, Shen XM, et al. Development and psychometric properties of the Chinese version of Children’s Sleep Habits Questionnaire. Zhonghua Er Ke Za Zhi. 2007;45(3):176–180. 17504618

[22] Sibley BA, Etnier JL. The relationship between physical activity and cognition in children: A meta-analysis. Pediatr Exerc Sci. 2003;15(3):243–256.

[23] Donnelly JE, Hillman CH, Castelli D, et al. Physical activity, fitness, cognitive function, and academic achievement in children: a systematic review. Med Sci Sports Exerc. 2016;48(6):1197–1222. doi: 10.1249/MSS.0000000000000901 27182986 PMC4874515

[24] ActiGraph. What is MVPA and how I can view in ActiLife. https://help.theactigraph.com/entries/22148365-What-is-MVPA-and-how-can-I-view-it-in-ActiLife. Accessed 6th September, 2017.

