# Supplementary material for: Study protocol for a randomized controlled trial comparing the effectiveness of physical exercise and melatonin supplement on treating sleep disturbance in children with autism spectrum disorders
Source: PLoS One. 2022 Jul 6;17(7):e0270428. doi: 10.1371/journal.pone.0270428 (PMC9258841; doi:10.1371/journal.pone.0270428)
Supplement: S1 File — (PDF) [file pone.0270428.s002.pdf]

**Reference Number**	2019-2020-0470
**State**	Reply To PI
**User ID**	andytcy
**Name of Investigator**	TSE Choi Yeung, Andy
**Post**	HPE - Assistant Professor
**Telephone Number**	29488074
**Telephone Number**	29488074
**Type of Funding**	External Funding
**Level/Category**	Other
**Title of the Funding Source**	HMRF
**Project Title**	Comparing the effectiveness of physical exercise and melatonin supplement in treating sleep disturbance in children with ASD: A RCT study
**Project From**	9/1/2021
**Project To**	6/30/2023
**Area/Field of Research**	Sports medicine

Purpose of the Research
2-3 sentences explaining the main goal of the research

The aim of the study is twofold: (1) To determine which intervention is more effective in improving sleep quality in children with ASD and 2) to explain how these interventions impact on sleep via melatonin-mediated mechanism model.

Prior to the study, information about the study will be provided to all participants and their parents. Written consent and verbal assent will be obtained from them. All data will be encrypted with passwords and only the PA and his delegates will have access to the data sets Ethical approval will be obtained from the PI's institution and the proposed protocol will be registered as a clinical trial.

Each participant will attend 2 one-week-long assessments in their respective schools, where we will assess their habitual sleep patterns and endogenous melatonin level before the intervention (T1), in the mid of the study (5 weeks after the commencement of the study) (T2) and after the 10-week intervention (T3). The mid-assessment is valuable to know how much variance in sleep behaviors is accounted by variance in melatonin, as well as to assess the adherence of the interventions.

Intervention A - Jogging group: The training protocol, assessment and instructional technique will be referenced on our previous protocol in children with ASD. However, we will modify the frequency of the jogging sessions to suit the comparison with other intervention group (i.e. intervention B). This intervention will be a 10-week jogging program consisting of 50 sessions (5 sessions per week, 30 min per session) in each participating school. The increase of jogging sessions from the previous protocol is to make the prescription equivalent to the melatonin supplement prescription for the intervention B, which will be prescribed to the participants every day, according to the previous study. Moreover, this is more close to the World Health Organization (WHO) recommendation that everyone should participate in physical exercise daily. Meanwhile, the jogging program is confined to morning sessions based on the favourable sleep outcomes from previous study. **To counteract the possible influence of natural sunlight exposure, all jogging sessions will be confined to indoor setting.** Each intervention session will be administered by a trained research assistant assisted by student helpers. All the research staff (i.e. student helpers and research assistant) will be required to attend a workshop (see educational plan) to standardize the procedure on implementing the intervention, as well as handling and motivating their ASD partners. Each session will be conducted in an identical format with 5 minutes of warm-up activities, followed by 20 minutes of jogging (intervention), and 5 minutes of cool-down activities. In the jogging activity, participants will be asked to jog side-by-side with the research staff around an activity circuit (57m x 50m) marked with 4 red cones. Participants are instructed to jog at a moderate intensity level. The intensity level of jogging will be measured by heart rate monitor (Polar H1). Considering the general low physical fitness of children with ASD, physical exercise with a heart rate above 50% of the maximum heart rate (subtracting the participant's age from 220) is considered to be moderate intensity. Motivation techniques (e.g. verbal cue, visual chart, favourite post-intervention activity) in previous protocol will be used to enhance the compliance rate. Meanwhile, questionnaire will be given to the research staff assisting the jogging intervention to assess the adherence of the intervention at T2. The feasibility, community-viability and sustainability of the exercise intervention are confirmed with the completed pilot study and the cooperation of the participating schools. Since these intervention sessions are conducted in the morning hours before school start times, the participants may get less sleep time since they have to arrive at school early enough to do the exercise. To have the same amount of time in bed, they are strongly encouraged to go to bed earlier at night.

Intervention B – Melatonin supplement group: Participants in this intervention group will undergo a 10-week melatonin supplement intervention period, where melatonin supplement (Natrol®, Chatsworth CA) will be provided 30 minutes before bedtime. The prescription time (i.e. 30 minutes before bedtime) and the dosage of 3mg will be used because these are optimal for most of the participants as suggested by Malow and colleagues. According to our Co-A, 1 mg and 9 mg are not suggested to ensure the effectiveness of the intervention while preventing the potential daytime sleepiness. Similar to the aforementioned intervention, questionnaire will be sent to parents to assess the adherence of the intervention at T2.

Intervention C – Combination group: Participants will receive the jogging program and supplemental melatonin dose with identical format as that in the intervention A and B (e.g., identical duration, identical manpower, identical warm-up and cool-down, identical dose, identical acclimation procedure before the intervention). Questionnaire will be sent to both research staff and parents to assess the adherence of the intervention for this group at T2.

Placebo control group: Participants in the placebo control group will receive no jogging and melatonin supplement dosing activity. However, they will be given a placebo flavored similar to the melatonin supplement (compounded by Pharmacare, Mt. Juliet, NT®). Meanwhile, they will also be required to wear an actigraph to control for their physical activity level at the assessment points (i.e. T1, T2, and T3). They will be expected for following their daily routine without participating in any additional formal physical exercise

training program throughout the whole study period (T1-T3). After T3, they will be assisted with jogging program to recognize their contribution as controls.

Remarks of the assessment

For the sleep log assessment, it will take approximately 20 minutes for parents to fill in the sleep log during each day. For the urinary sample collection, it will take approximately 1 minute for each time. All the assessments and interventions will not be video-recorded.

Sample size estimation

Previous study showed physical exercise had notable effects (corresponding to a Cohen's *d* of about 0.9) on improving sleep onset latency, sleep duration and sleep duration. We assumed that melatonin supplements have similar effects on sleep. A sample of 20 participants per group is required to achieve a power of 80% and a level of significance of 5%. Assuming 20% attrition rate, 25 participants per group will be recruited (i.e. the total number is 100).

Randomization

Participants will be randomly assigned to three intervention groups or a placebo-control group. Block randomization with block size of 5 will be used for equal allocation ratios.

Blinding

The person analysing the sleep parameters and melatonin level will be blinded for the group assignment.

Does your research involve human participants directly?

Yes

Does your research involve other human data, e.g. secondary data, archival data, etc.?

No

Please fill in the below information about the participants (in groups) involved in your research project including number of participants, backgrounds of the groups and age range, etc.

Potential subjects will be recruited from at least four local special schools for mild intellectual disabilities that agreed to join the research project. The special schools are preferred because most of the children with ASD in Hong Kong are studying in the special schools in Hong Kong. They will be screened with the following inclusion and exclusion criteria. The target number of participants is 100.

The inclusion criteria are: (1) clinical diagnosis of ASD by a physician or clinical psychologist based on the Diagnostic and Statistical Manual of Mental Disorders, 5th edition, (DSM-5) and confirmed with the Autism Diagnostic Interview- Revised (ADI-R); (2) clinical diagnosis of sleep disturbance by a physician; (3) age 8 – 10 years (to keep consistent with our pilot study and previous published study protocol[18]); (4) pre-puberty as indicated by Tanner stage I through screening by a physician (to prevent any puberty influence on hormonal response); (5) being given an average of 8 hours of sleep per night by their parents over the past 3 months; (6) parents reported sleep onset delays of 30 minutes of longer on three or more nights per week; (7) free of psychotropic medications (allergy medications and medications for constipation are allowed); (8) non-verbal IQ over 60 using a brief version of Wechsler intelligence scale for children (Chinese revised, C-WISC) (since lower IQ is liked to higher prevalence of comorbid psychiatric disorders and seizures); (8) Social Response Scale T-score 80 or above (to control for the ASD severity) and (9) able to perform the requested physical intervention.

Exclusion criteria are: (1) with one or co-morbid psychiatric disorders identified with a structured interview based on DSM-5; (2) with other medical conditions that limit their physical exercise participation and sleep (e.g., asthma, seizure, cardiac disease etc); and (3) with a complex neurologic disorder (e.g., epilepsy, phenylketonuria, fragile X syndrome, tuberous sclerosis) and (4) participants who are currently meeting physical exercise guidelines (i.e. 60 minutes of moderate and vigorous physical exercise each day). All screening will be carried out by clinical psychologists coordinated by Dr. Esther Lau (the Co-A), Dr. Lai Wing Him (psychiatric fellow, the Co-A) and Dr. James Cheng (Paediatrics Resident, the Co-A). In addition to this formal screening, we will collect information for each participant from the parents, including records in after-school group therapy (e.g. occupational therapy, speech therapy) and medication usage. After screening, parents will be instructed to maintain the normal routine physical exercise pattern and sleep pattern of their children throughout the study period.

Are there any reimbursements or other incentives to participants?

Yes

Please mention the cost and the form of reimbursements or incentives offered and clarify why they are reasonable)

$300 supermarket coupon will be distributed to each participant's family to recognize their efforts in participating in this study.

Please explain your way(s) of recruiting your participants and inviting them to join in step-by-step detail.

Potential subjects will be recruited from at least four local special schools for mild intellectual disabilities that agreed to join the research project. The special schools are preferred because most of the children with ASD in Hong Kong are studying in the special schools in Hong Kong.

Information about the study will be provided to all participants and their parents. Written consent and verbal assent will be obtained from them.

**If applicable, explain how you will obtain the participants' contact information in detail.**	Written consent, contact information and verbal assent will be obtained from them. All data will be encrypted with passwords and only the PA and his delegates will have access to the data sets Ethical approval will be obtained from the PI's institution and the proposed protocol will be registered as a clinical trial.
**Does your research project involve organizations other than EDUHK?**	Yes
**Please identify the data collection site(s), and describe how you will obtain consent/ permission from the data collection site(s). If no written consent will be obtained, please state the reasons**	Potential subjects will be recruited from at least four local special schools for mild intellectual disabilities that agreed to join the research project. The special schools are preferred because most of the children with ASD in Hong Kong are studying in the special schools in Hong Kong. Information about the study will be provided to all participants and their parents. Written consent and verbal assent will be obtained from them. All data will be encrypted with passwords and only the PA and his delegates will have access to the data sets Ethical approval will be obtained from the PI's institution and the proposed protocol will be registered as a clinical trial.
**Are there any relationships between people involved in the recruitment and the participants (such as teacher and students, principal and teachers, nurse and patients)?**	Yes
**State the nature of the relationship, and mention the special precautions which will preserve their rights to decline to join or withdraw from participation once the research has started**	Teachers and the students. All the teachers, participants and their parents will be briefed by the research team that the participation of the study is on voluntary basis and that they can withdraw from the study without any consequence.
**NotAbleToGiveConsent**	Yes
**b. Will there be any coercion on the part of the investigator?**	No
**c. Will the data collected have any personally identifiable information of living people, such as name, address, ID numbers, etc? (If so, see HREC Operational Guidelines - Part V regarding Confidentiality and Storage of Data)**	Yes
	No
**e. Will the study collect information regarding sensitive aspects of the research participants' behavior such as drug and alcohol use, illegal conduct, or sexual behavior?**	No
**f. In case the information on the research participants is disclosed, will it reasonably place the research participants at risk of civil or criminal liability or damage the research participants' financial standing, employability or reputation?**	No
**g. Will financial or other inducements (other than reasonable expenses and compensation for time) be offered to research participants?**	No
**h. Will deception of research participants be necessary during the study? (If so, explain why deception is necessary. Also, please include information on debriefing procedures)**	No
**i. Will the study involve prolonged and repetitive testing?**	No
**j. Will the study cause psychological stress or anxiety?**	No

**k. Will pain or more than mild discomfort is likely to result from the study?**	No
**l. Are drugs or placebo to be administered to the research participants?**	No
**m. Will the study involve any intervention?**	Yes
**n. Will blood or tissue samples be obtained from research participants?**	No
**o. Will the research involve any DNA work or human embryo or stem cell research?**	No
**p. Will the research participant's identity be disclosed if archived tissue samples or personal / medical / social records are used? If your study is not a medical/clinical research, please choose NA.**	No
**q. Will you use irradiation or hazardous substances on research participants?**	No
**r. Will the study impinge on the research participants' right to privacy or their personal life?**	No
**s. If you have checked "Yes" to any of the above questions, please provide elaboration below:**	a. Subjects Potential subjects will be recruited from at least four local special schools for mild intellectual disabilities that agreed to join the research project. The special schools are preferred because most of the children with ASD in Hong Kong are studying in the special schools in Hong Kong. They will be screened with the following inclusion and exclusion criteria. The inclusion criteria are: (1) clinical diagnosis of ASD by a physician or clinical psychologist based on the Diagnostic and Statistical Manual of Mental Disorders, 5th edition, (DSM-5) and confirmed with the Autism Diagnostic Interview- Revised (ADI-R); (2) clinical diagnosis of sleep disturbance by a physician; (3) age 8 – 10 years (to keep consistent with our pilot study and previous published study protocol[18]); (4) pre-puberty as indicated by Tanner stage I through screening by a physician (to prevent any puberty influence on hormonal response); (5) being given an average of 8 hours of sleep per night by their parents over the past 3 months; (6) parents reported sleep onset delays of 30 minutes of longer on three or more nights per week; (7) free of psychotropic medications (allergy medications and medications for constipation are allowed); (8) non-verbal IQ over 60 using a brief version of Wechsler intelligence scale for children (Chinese revised, C-WISC) (since lower IQ is liked to higher prevalence of comorbid psychiatric disorders and seizures); (8) Social Response Scale T-score 80 or above (to control for the ASD severity) and (9) able to perform the requested physical intervention. Exclusion criteria are: (1) with one or co-morbid psychiatric disorders identified with a structured interview based on DSM-5; (2) with other medical conditions that limit their physical exercise participation and sleep (e.g., asthma, seizure, cardiac disease etc); and (3) with a complex neurologic disorder (e.g., epilepsy, phenylketonuria, fragile X syndrome, tuberous sclerosis) and (4) participants who are currently meeting physical exercise guidelines (i.e. 60 minutes of moderate and vigorous physical exercise each day). All screening will be carried out by clinical psychologists coordinated by Dr. Esther Lau (the Co-A), Dr. Lai Wing Him (psychiatric fellow, the Co-A) and Dr. James Cheng (Paediatrics Resident, the Co-A). In addition to this formal screening, we will collect information for each participant from the parents, including records in after-school group therapy (e.g. occupational therapy, speech therapy) and medication usage. After screening, parents will be instructed to maintain the normal routine physical exercise pattern and sleep pattern of their children throughout the study period.<br><br>c. Contact information of the participants and parents will be obtained so as to identify which urinary samples belonged to whom. The contact information will also be used for delivery of summary report to the participants and their parents.<br><br>m. Participants in the placebo control group will receive no jogging and melatonin supplement dosing activity. However, they will be given a placebo flavored similar to the melatonin supplement (compounded by Pharmacare, Mt. Juliet, NT®). Meanwhile, they will also be required to wear an actigraph to control for their physical activity level at the assessment points (i.e. T1, T2, and T3). They will be expected for following their daily routine without participating in any additional formal physical exercise training program throughout the whole study period (T1-T3). After T3, they will be assisted with jogging program to recognize their contribution as controls.<br>NA<br>Children may feel thirsty during jogging sessions, water will be provided during exercise interventions.<br><br>A few studies have investigated the safety of melatonin, but none have revealed any serious side effects. It also doesn't seem to cause any dependence or withdrawal symptoms

A few studies have investigated the safety of melatonin, but none have revealed any serious side effects. It also doesn't seem to cause any dependence or withdrawal symptoms.[1-2]

[1]Andersen LP, Gögenur I, Rosenberg J, Reiter RJ. The Safety of Melatonin in Humans. *Clin Drug Investig*. 2016;36(3):169-175.

[2]Lyseng-Williamson KA. Melatonin prolonged release: in the treatment of insomnia in patients aged ≥55 years. *Drugs Aging*. 2012;29(11):911-923.

The study will only be launched when there is no government warning signal for COVID-19. Moreover, to ensure safety of each personnel in this project, all the participants and research staff will be wearing surgical masks when implementing the intervention and administering data collection, as well as keeping the social distance of 1.5m from each other.
All data will be encrypted with passwords and only the PA and his delegates will have access to the data sets Ethical approval will be obtained from the PI's institution and the proposed protocol will be registered as a clinical trial. All data will be stored for one year in PI's office computer and they will all be destroyed one year after the completion of the study.

c. Other than the above (3a/3b), will the results or data be given to any other persons (e.g., shared on an open-science website) or organizations? No

c. Other than the above (3a/3b), will the results or data be given to any other persons (e.g., shared on an open-science website) or organizations? No

Co-Investigator

Full Name (Last Name First)	Post (please also state respective institution here for external member)	Department (for EdUHK staff only)	Tel No	Email
Lee Paul Hong	Assistant Professor, Hong Kong Polytechnic University		34008275	paul.h.lee@polyu.edu.hk
Ho Amy Wing Yin	Medical Technologist, The Chinese University of Hong Kong		35052082	hwy104@ha.org.hk
Cheng James Wesley Ching-hei	Associate Consultant, United Christian Hospital		39496109	cch278@ha.org.hk
Lai Wing Him Elvis	Associate Consultant, Castle Peak Hospital		60109601	lwh041@ha.org.hk
Lau Esther Yuet Ying	Assistant Professor	PS	29488253	laueyy@eduhk.hk

Attachment

File	Description
HMRF_Invitation_Principal.doc@!@application_msword	Draft informed consent form/ invitation letter/ approval letter for data collection sites (if applicable), (e.g. school)
Appendix 2 Sleep log assessment form.pdf@!@application_pdf	Data collection form, including questionnaire (if applicable)
HMRF_Proposed_Research_Project_2019.pdf@!@application_pdf	Research Proposal
HMRF201920_Informed cosent_parents_participant.pdf@!@application_pdf	Consent form and Information Sheet for Parents
